# Female genital mutilation: trends, economic burden of delay and basis for public health interventions

**DOI:** 10.1186/s12939-024-02140-4

**Published:** 2024-04-15

**Authors:** Kathya Cordova-Pozo, Hisham Hussein Imam Abdalla, Ann-Beth Moller

**Affiliations:** 1 Institute for Management Research, Radboud University, Nijmegen, The Netherlands; 2 Department Obstetrics and Gynecology, Faculty of Medicine, Minia University, Minia, Egypt; 3 School of Public Health and Community Medicine, Institute of Medicine, Sahlgrenska Academy, University of Gothenburg, Gothenburg, Sweden

**Keywords:** Female genital mutilation, Sustainable development goals, Effective interventions, Economic burden of delay, Economic burden of non-abandonment, Harmful practices

## Abstract

**Background:**

The practice of female genital mutilation (FGM) is a health and social problem. Millions of girls and women have undergone FGM or will soon, and more information is needed to effectively reduce the practice. The aim of this research is to provide an overview of the FGM trendlines, the inequality of its prevalence, and the economic burden. The findings shed light on 30-year trends and the impact of the pandemic on planned efforts to reduce FGM which helps with public health interventions.

**Methods:**

Temporal trend analysis, and graphical analysis were used to assess the change and inequality over the last 30 years. We included 27 countries in which FGM is prevalent. We calculated the extra economic burden of delayed interventions to reduce FGM like COVID-19.

**Results:**

For the 27 countries analyzed for temporal trendlines, 13 countries showed no change over time while 14 had decreasing trends. Among the 14, nine countries, Uganda, Togo, Ghana, Benin, Kenya, Nigeria, Central African Republic, Chad, and Ethiopia had high year-decrease (CAGR − 1.01 and − 10.26) while five, Côte d’Ivoire, Egypt, Gambia, Djibouti, and Mali had low year-decrease (CAGR>-1 and < 0). Among these five are the highest FGM prevalence similar distribution regardless the wealth quintiles or residence. There is an economic burden of delay or non-decline of FGM that could be averted.

**Conclusion:**

Findings indicate that some countries show a declining trend over time while others not. It can be observed that there is heterogeneity and homogeneity in the FGM prevalence within and between countries which may indicate inequality that deserves further investigation. There is considerable economic burden due to delays in the implementation of interventions to reduce or eliminate FGM. These insights can help in the preparation of public health interventions.

**Supplementary Information:**

The online version contains supplementary material available at 10.1186/s12939-024-02140-4.

## Plain summary

The practice of female genital mutilation (FGM) is a health and social problem reflecting a deep-rooted practice. More insights are needed to improve the effectiveness of public health interventions in their effort to reduce FGM. This study evaluated the progress of FGM in the past 30 years, the inequality of its prevalence, and the economic burden exacerbated by COVID-19. The findings show that there are important economic burden costs due to pandemic or delay of non-abandonment in the prevalence of FGM.

## Background

Female genital mutilation (FGM), also known as cutting or circumcision, but the preferred term for the World Health Organization (WHO) is FGM as it involves a form of mutilation, a permanent partial or complete removal of external genitalia or other injury for non-medical reason [1]. There are four types. *Type I, clitoridectomy*, involves the partial or total removal of the clitoris; *Type II, excision*, involves the partial or total removal of the clitoris and the labia minora, with or without excision of the labia majora; *Type III, infibulation*, involves narrowing of the vaginal opening through the creation of a covering seal; *Type IV, all other harmful procedures*, e.g., cauterizing [1–3]. About 200 million girls suffered FGM before adolescence in 27 countries which is mostly carried out by traditional practitioners (82%), and 18% by health care providers but with an increasing trend [2, 3]. Data exists for 27 countries but FGM is practiced in many others around the world with less intensity in comparison with these 27 countries [3, 4]. FGM increases the risk of health complications and costs of treatment throughout the life course and violates human rights [5, 6]. The Sustainable Development Goals (SDG), target 5.3 on gender equality, aims to eliminate harmful practices by 2030, FGM being one of them [4] and reducing inequality within and among countries (SDG 10).

The practice of FGM is a health and social problem that reveals deep-rooted inequality between the sexes, harmfully affecting women, but also it reveals differences between and within countries. Various social reasons maintain the practice such as signal of chastity and fidelity in well-stratified polygynous societies [7, 8], increasing daughter’s value for marriageability and attracting wealthy husbands [2, 8, 9], and the social value of the practice as a baptism [7, 9, 10]. Many public health interventions have been established to eliminate the practice [11], such as legislation to criminalize the practice or anti-FGM campaigns or education, but eradication is hindered by political commitment and continuous investment to eliminate the practice [4, 12]. To understand the effectiveness of interventions [2, 13, 14], it is worth noting that research and interventions focused on the practice as the countries where it is practiced were the same, ignoring particularities in each of this countries or the reasons behind the practice [3, 9, 13]. However, the gap lies in understanding better the practice of FGM within its specific country. With the current prevalence in these 27 countries, the current state of the art calculates an economic burden for FGM is 1.4 billion USD per year projected to rise to 2.1 billion by 2047 [4]. Economic burden includes all the yearly costs associated with the treating a wider range of health conditions associated with FGM. Hence, more evidence is needed to monitor the reduction of FGM prevalence rates as well as the reduction of economic burden in these countries through time and gain insights for improving scale-up interventions [2, 13] in terms of which interventions effectively reduce the prevalence and which are the main barriers [15]. For instance, there are some qualitative studies indicating that COVID-19 affected the health interventions and the allocation of resources because of the lockdowns [11, 16] but not much is known about the economic burden caused by COVID-19 because the interventions were stalled and the projected abandonment could not occur. This is what we call economic burden of delay. Hence, this research will build on top of the state of the art [4] to calculate the impact of delay. The aim of this research is to provide an overview of the FGM trendlines, the inequality of its prevalence, and the economic burden exacerbated by COVID-19. The findings shed light on the progress over the past 30 years and the impact of the pandemic on planned efforts to reduce FGM which shapes future health interventions and scale-up strategies.

## Methods

To gain a deeper understanding of the persistence of FGM, and address the associated economic burden, we used a longitudinal research design using FGM prevalence data of the past 30 years, 1990–2020, and prospective analysis with economic burden analysis, 2020–2047 using different sources of data. For the trend analysis and the inequality assessment, we obtained indicators of FGM prevalence among girls and women aged 15 to 49 years, prevalence by areas of residence (rural, urban) and wealth quintiles covering the period 1990–2020 from the Demographic and Health Surveys (DHS) [17]. We also retrieved data with estimates of prevalence for year 1990 from World Health Organization (WHO)^1^ [18]. For the economic burden analysis, we applied the parameters and assumptions used by Tordrup D. et al. (2022) as it includes the same set of countries as used for this study [4].

To obtain knowledge that can support future health interventions, we proposed different key analysis, (1) trend analysis, (2) inequality, (3) economic burden of delay. We conducted a temporal trend analysis using a linear regression of FGM prevalence indicating the general tendency across 27 countries with three or more datapoints. The trend-results were used to classify countries in decreasing, increasing, or no change using a *p*-value ≤ 0.1 [19] and a R²≥0.5 as an indicator of linear dependance and reliability [20], which means that the time series can show a pattern for the trend. We also calculated the average year-change of the FGM prevalence using a compound annual growth rate, formula 1 (CAGR) [21]. The CAGR is used to have the average year-change similar to the compound interest on a savings account and is very much used in finance [21, 22] but it starts to be more used in health as well [22, 23] because it shows a year progress instead of an absolute number, and it can complement the trendline analysis to draw better conclusions reducing the bias with either of these alone [12, 19]. This is particularly useful when we want to observe how much FGM progressed yearly and a way of quantifying it over a specific period while a regular average growth rate has a linear approach. To detect high (CAGR<-1) and low (CAGR>-1 and < 0) shifts per year-decrease which help to understand the progression or trends.

1 $$ CAGR\left( \% \right) = \left( {{{\left( {\frac{{end\_value}}{{start\_value}}} \right)}^{\left( {\frac{1}{t}} \right)}} - 1} \right) \cdot 100\% $$

Where:

Start and end value = represent the year prevalence at different points in time.t = number of years

To analyze inequality, per country and per group of countries, we used the FGM prevalence divided by residence and wealth quintile [17] from DHS. This means, we made graphs to visualize the FGM prevalence according to the level of wealth (from poorest to richest) or residence in rural or urban areas. The graphs portray inequality between and within countries helping exploring the problem differently, particularly when comparing with the trend analysis and later with the economic burden analysis.

The economic burden analysis due to delay. was calculated by comparing a delay scenario with two baseline scenarios where we assumed a hypothetical abandonment of 100% or full abandonment and 50% or partial abandonment the FGM practice. The difference of yearly FGM prevalence in every scenario was multiplied with the cost per case to define the yearly burden. We focused on the extra costs that originate from two types of delay: a)*the 3-year COVID-19 delay*, as the lockdowns stalled programs to stop the FGM [11, 16], b) *the delay due to non-abandonment*, as there are countries where trends indicate where the FGM prevalence rate is stable [4]. For the first, we used 0% abandonment during 2020–2023, meaning that the prevalence in 2019 would not change for three years except for natural population growth. The extra costs due to the delay of the pandemic were estimated by comparing the prevalence with the alternative 100% and 50% abandonment. The second: *delay due to non-abandonment*, estimates the economic burden of maintaining the prevalence rate is stable through the years using Somalia as an example. We used a FGM related cost ranging ranges from 10.37 to 12.46 USD per prevalent case per year [4]. We used the conservative estimation of 10.37 USD, which reflects FGM related costs and includes healthcare costs for treating health conditions that may arise from FGM. We used R version 4.2.1, and Microsoft Excel 365 version 2309 for the analysis.

## Results

Of the 27 countries included in the analysis, 13 countries showed no significant trend over time (*P*-value > 0.10), while 14 had significant decreasing trends (*P*-value < 0.10) (Appendix 1). Between the 14 countries with decreasing trends, low and high year-decrease were identified through CAGR. Five countries, Côte d’Ivoire, Egypt, Gambia, Djibouti, and Mali had low year-decrease (CAGR >-1% and < 0%) while nine had high year-decrease (CAGR < -1%), Uganda, Togo, Ghana, Benin, Kenya, Nigeria, Central African Republic, Chad, and Ethiopia. The largest year-decrease was in Uganda (-10.26%), and the lowest was Ethiopia (-1.01%), see Table 1. It is worth mentioning that even if there is no significant trend countries could have CAGR indicating year-changes. This is the case for Niger that reduce the prevalence considerably with a CAGR of -9.94% (See Appendix 2) but doesn’t show a trendline with a significant *P*-value, the same as the other five countries presented in Table 1 with no trends.

**Table 1:** High-low and no trends of FGM prevalence rates, *p*-value ≤ 0.10, and its compound annual growth rate, percentages, 1990–2020

Country	Income category	Start prevalence rate (%)	Start year	Last prevalence rate (%)	End year	Significance (*P*-value < 0,10)	CAGR (%)
**High decreasing rates: 5 countries with the highest**							
Uganda	Low	5	1990	0,3	2016	*	-10,26
Togo	Low	50	1990	3	2017	*	-9,79
Ghana	Low-middle	30	1990	2,4	2018	*	-8,63
Benin	Low-middle	50	1990	9	2014	*	-6,81
Kenya	Low-middle	50	1990	21	2014	*	-3,55
**Low decreasing rates: 5 countries with the lowest**							
Mali	Low	94	1990	89	2018	*	-0,21
Djibouti	Low-middle	98	1990	90	2019	*	-0,29
Gambia	Low	80	1990	73	2020	*	-0,30
Egypt	Low-middle	97	1990	87	2015	*	-0,43
Cote d’Ivoire	Low-middle	43	1990	37	2016	*	-0,61
**No trends: 5 countries with the highest prevalence**							
Somalia	Low	98	1990	99	2020		0,03
Guinea	Low	60	1990	95	2018		1,64
Sudan	Low	89	1990	87	2014		-0,12
Sierra Leone	Low	90	1990	83	2019		-0,28
Eritrea	Low	90	1990	83	2010		-0,40

NOTE: The column Significance shows the result from the linear regression to show a trendline with a single asterisk used to express *P* ≤ 0.10. And CAGR stands for compound annual growth rate that is another measure to show an average year-change

### Inequality between and within the countries

The graphical analysis in Appendix 3 and 4, reveal that the FGM prevalence has two groups of countries (Table 2). The first group of 14 countries have an heterogeneous distribution of the FGM prevalence in the population, being the most affected the poorest quintiles, and the ones living in rural areas, while the richest or people living in the urban area have a lower prevalence which exhibit inequality within the country. Moreover, this group has a high year-decrease with CAGR values <-1% and significant trendlines with few exceptions. The second group of 13 countries shows a rather homogeneous distribution of FGM across the population regardless of wealth or residence observing a high FGM prevalence. Additionally, this group has a low year-decrease with CAGR values >-1% and < 0%, and mainly non-significant trendlines with few exceptions. In this group, we observe inequality for not reducing the FGM rates. (Table 2).

**Table 2:** Distinction of countries based on the inequality between and within countries of the FGM prevalence according to wealth and residence

Country	Start prevalence rate (%)	Start year	Last prevalence rate (%)	End year	Significance (*P*-value < 0,10)	CAGR (%)	Distribution of FGM prevalence divided by residence	
**First group. Heterogeneneous distribution of the FGM prevalence**							Urban	Rural
Benin	50	1990	9,2	2014	*	-6,81	5	13
Central African Republic	43	1990	21,6	2019	*	-2,35	12	28
Chad	60	1990	34,1	2019	*	-1,93	32	35
Côte d’Ivoire	43	1990	36,7	2016	*	-0,61	31	44
Ghana	30	1990	2,4	2018	*	-8,63	1	4
Kenya	50	1990	21	2014	*	-3,55	14	26
Liberia	60	1990	32	2020		-2,07	25	43
Mauritania	25	1990	64	2020		3,18	55	79
Niger	20	1990	2	2012		-9,94	1	2
Nigeria	40	1990	15	2020	*	-3,22	24	16
Tanzania	10	1990	10	2016		0,00	5	13
Togo	50	1990	3,1	2017	*	-9,79	3	4
Uganda	5	1990	0,3	2016	*	-10,26	0	2
Yemen	22,6	1997	18,5	2013		-1,24	17	19
**Second group. Homogeneous distribution of the FGM prevalence**							**Urban**	**Rural**
Burkina Faso	70	1990	68	2015		-0,12	69	78
Djibouti	98	1990	90	2019	*	-0,29	94	98
Egypt	97	1990	87,2	2015	*	-0,43	77	93
Eritrea	90	1990	83	2010		-0,40	80	85
Ethiopia	85	1990	65,2	2016	*	-1,01	54	68
Gambia	80	1990	73	2020	*	-0,30	75	67
Guinea	60	1990	94,5	2018		1,64	95	94
Guinea-Bissau	50	1990	52,1	2019		0,14	43	59
Mali	94	1990	88,6	2018	*	-0,21	89	88
Senegal	20	1990	25,2	2019		0,80	21	29
Sierra Leone	90	1990	83	2019		-0,28	76	89
Somalia	98	1990	99	2020		0,03	99	99
Sudan	89,2	1990	86,6	2014		-0,12	86	87

NOTE: For clarity, we only present the FGM distribution by residence and more details can be found in the Appendix 3 and 4

### Economic burden analysis

#### The impact of COVID-19

**Fig. 1:**
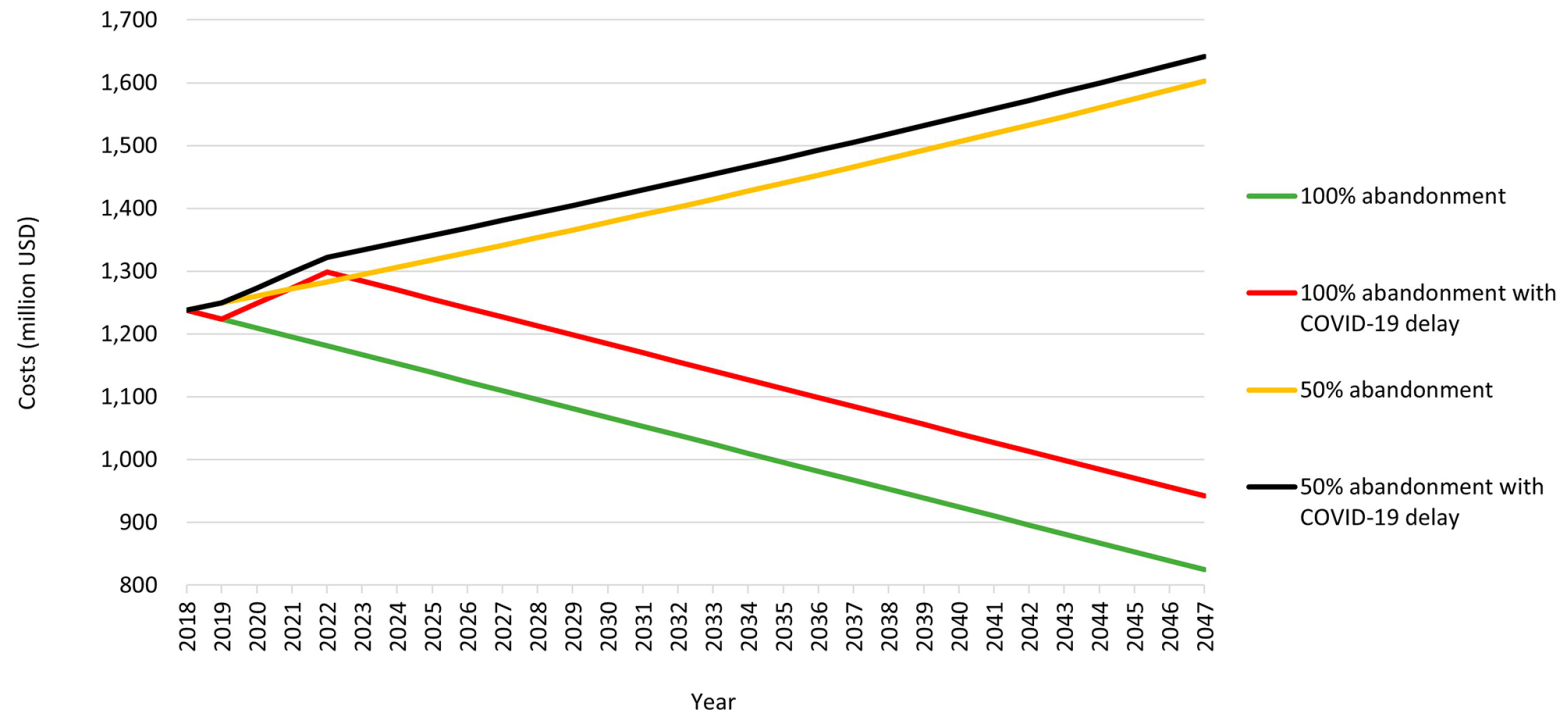
Economic burden of the delay in reducing FGM prevalence rates due to the COVID-19

Figure 1 shows the economic burden of FGM or the effects of a delay due to COVID-19 on the FGM prevalence and the costs for a 100% abandonment and 50% abandonment scenarios. Both scenarios show that the delay increases the prevalence during the COVID-19 period as it falls back to a 0% abandonment. After the pandemic, it follows the normal progress of interventions according to the scenario. The yellow and the green line represent the costs when abandoning FGM by 50% and 100% respectively under normal circumstances. The black and the red line represent the increased costs during COVID-19, because there was 0% abandonment for the 50% and the 100% abandonment. The calculation of the difference between these situations lead to an incremental cost of 679 USD million for a delay of 3 years in the scenario of 50% and an incremental cost of 2026 USD million for the scenario of 100%.

#### The costs of non-abandonment

**Fig. 2:**
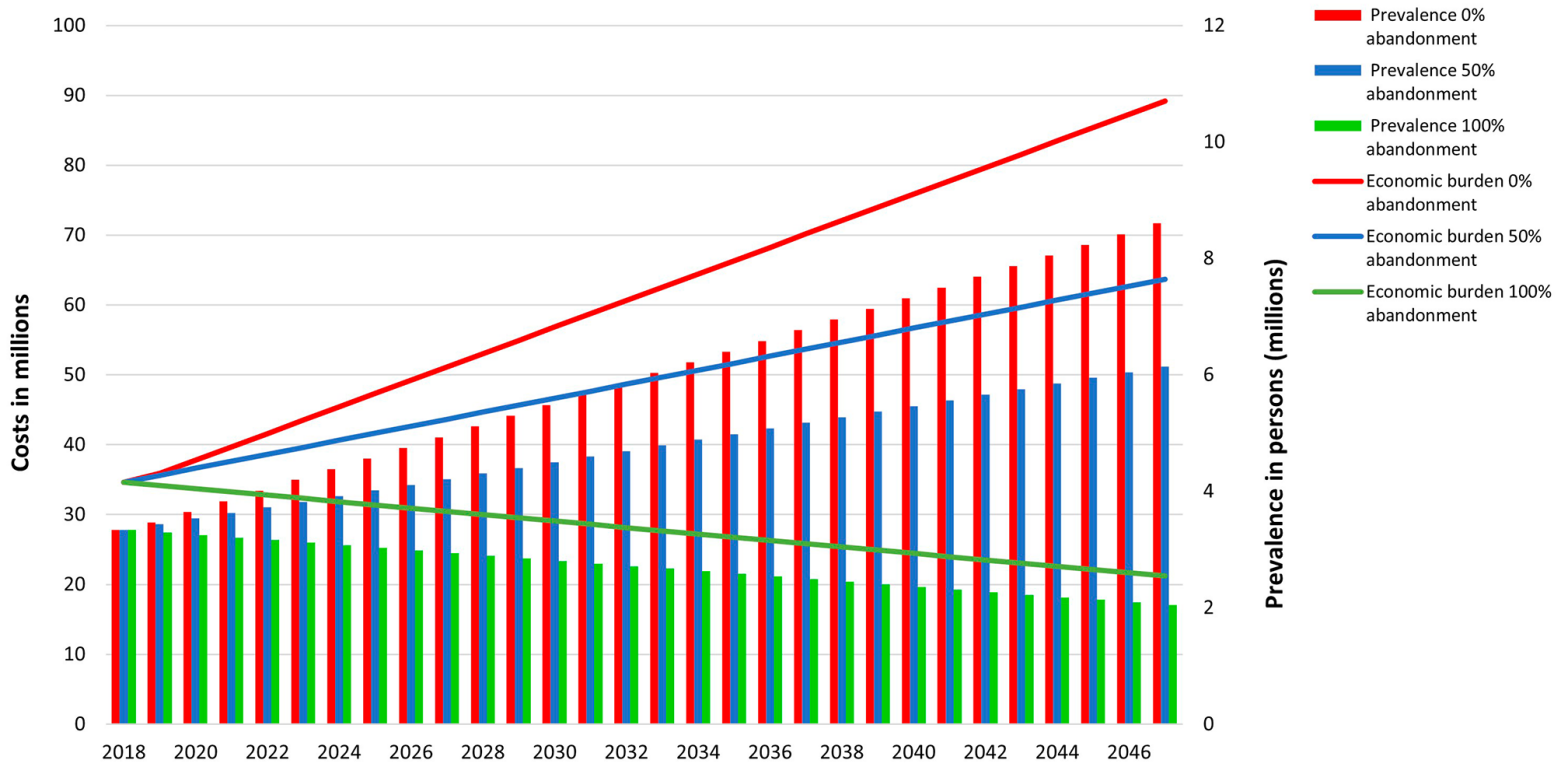
Somalia: Economic burden of the non-abandonment in the FGM prevalence rates

Figure 2 shows the economic burden and the prevalence of FGM over time for Somalia and for three levels of abandonment: 0%, 50% and 100%. Abandonment of 0% and 50% let the prevalence and the economic burden grow over time. This is partly also due to population increase. An abandonment of 100% allows the prevalence reduce over time. We can evidence that having no abandonment increase the prevalent cases creating an extra economic burden. Somalia could avert 212 USD million by 2047 in a scenario of 50% abandonment, and 576 USD million in the scenario of 100% abandonment. The economic burden for FGM per year accounts for 1.65% of current health expenditure of Somalia^2^.

## Discussion

The findings show that reducing the FGM prevalence can avert the economic burden, particularly when observing all the additional costs exacerbated due to COVID-19 causing a delay in the progress toward elimination of FGM because interventions were stalled. Of all 27 countries analyzed for temporal trends, 13 countries showed no significant trend over time (*P*-value > 0.10), while 14 had significant decreasing trends (*P*-value < 0.10). Among the countries with decreasing trends, some experienced a low year-decrease while others had a high-decrease over time. High year-decrease is found among the countries with low FGM prevalence while low year-decrease is within the countries with high prevalence, e.g., Mali, Djibouti, and Gambia. Findings show that these two groups of countries show inequality in different ways. First, **within***countries with heterogeneous distribution*, as the poorest quintiles or the ones living in the rural areas are the most affected with FGM while richer quintiles or people living in the urban areas are not. Second, inequality in the decline of the FGM prevalence as they maintained stable rates over time and this creates inequality **between***countries with in the first group)* and *the second group* because this last did not decline its prevalence rates within 30-year time while the first did.

Findings show the importance of distinguishing countries that practice FGM. This distinction may help planning of interventions within the needs of the country. Systematic literature reviews (SLR) indicate a list of effective interventions that include participatory action research, multisectoral approach, and long-term planning have all together an effect as it considers the enablers and barriers permeating the entire society like education-based interventions, including training, and empowerment [13, 24, 25]. The SLR identify shortcomings like the lack of involvement of men, the absence of a multisectoral approach, or just collecting surveys about the health belief model which are not effective to demonstrate change [2, 13, 14]. This study shows that it is important to compare the interventions against changes in outcomes of FGM practice, and maybe observe the particularities of the practice within the wealth-quintiles or place of residence. The first argument is in line with other authors [4], and further studies are needed for deeper analysis on the differences within and between countries.

Based on the findings, it is important to recognize that the practice originates in the values of the community. However, most of the preventive interventions were focused on the negative consequences of the practice, and punitive actions to establish fear but no recognition to change the practice from the values it entails. Moreover, in some countries, it can be observed that if interventions are targeted to reducing wealth disparities, the practice may reduce while the ones not influenced by wealth may need other types of intervention. This is evidenced in the outcomes of Kenya with a high year-decrease (CAGR = 3.55) and Egypt with a low year-decrease (CAGR = 0.43) in the past 21 years. Both countries had a similar sustained economic growth, with Kenya having a better income distribution than Egypt [26]. Kenya identified four barriers: (1) deeply rooted cultural beliefs, (2) medicalization, (3) lack of multilateral efforts, (4) hindered accessibility in remote areas for interventions and worked on them [10, 27]. Success can be attributed to a combination of factors, (1) legal framework that penalizes and protects from harmful practices, (2) educational programs for awareness, dialogue, and cooperation. Similarly, Egypt applied similar laws and actions as Kenya but the practice remains highly prevalent [9, 24]. Religious, social, and cultural factors may trigger this together with low education and rural residence. For instance, the society identifies FGM with purification, a milestone in the woman’s life, and the majority of men prefer to marry circumcised girls which is a social pressure [3, 9, 24]; an underlying support of the Muslim leaders despite the official condemnation. This could be comparable to Somalia, Sudan, Guinea, and Mali [3]. Other authors indicate that Egypt had a small and steady decline in all segments [28].

These outcomes suggest the need to separate them to prepare tailor-made interventions to tackle specific aspects of the practice. Other authors indicated heterogeneity between rural vs. urban too [29]. Sustained and equal economic growth with increased quality of life can support reducing the prevalence among the countries with heterogeneity in the FGM practice.

For the countries that have homogeneity, interventions need a new thinking of multilateral and multisectoral approach that include other aspects such as culture and values of the society or other to effectively eliminate the FGM. There are at least three theories that could help tailoring new interventions. *First*, is the transtheoretical model or stages of change model indicating that behavioral change is a cyclical process and the different stages are influenced by different factors, and at different levels it needs stimulus control, and supportive environment [30]. Comprehensive monitoring is also important to adapt the content of the intervention [31, 32] to meet the stages of change [33, 34]. This approach has been used by multiple interventions such as reproductive health in adolescents [35], physical activity [36]. *Second*, the habit of persistence theory indicates that there are certain practices that are strongly embedded in the belief system and appertains to their deep identity keeping the practice over time and is hard-wearing. Marketing uses the approach based on the underlying values to motivate consumer behavioral change or help in the construction of technological frames to encompass development [37, 38]. *Third*, the means-end chain analysis that indicates that identity can be modified when there is a deep understanding of the means-end chains that seeks to understand the values underneath the actions of people facilitating marketing strategies for realizing the desired end states [39]. These theories may also explain why FGM is practiced despite the law regulations, the level of development, or place of residence. For instance, FGM is still practiced among people that migrated to higher income countries [25].

The literature revision shows that COVID-19 affected the interventions to reduce FGM with a delay. Workshops in schools or activism to increase social development were stalled or even diverted due to the COVID-19 lockdowns [11, 16]. Our analysis shows that a delay of 3 years to reduce the practice of FGM is costly, with an extra of 679 million USD in the scenario of 50% abandonment and 2026 USD million in the one of 100%. The case of Somalia also shows the costs of non-decline toward the elimination of the practice, showing that per year can be compared to 1.65% of the total health expenditure per capita. The extra costs of economic burden could be spent on economic development or other medical treatments. Somalia for example could avert 212 USD million by 2047 in a scenario of 50% abandonment, and 576 USD million in the scenario of 100% abandonment by 2047. Delay can also create behavioral regression that could even increase FGM practices [11, 16].

### Strengths and limitations

The strengths of this research are the broad analysis, the categorizing, the economic burden of delay for new theoretical and practical insights for interventions. First, the analysis is done through different lenses of statistical and case-analysis. Broad assessment and categorization can help prepare effective public health interventions. This study also has limitations. Ideally, this study would have included all the countries that practice FGM but lack of consistent data points and disaggregated data by age group (10–14, 15–19, 20–24) allowed us to only include 27 countries in the analysis. Still, it is important to consider that although trends in the prevalence can be analyzed, the data contains some limitations like the range of variations between years and countries, and number of data points [17], or that statistics may not reflect today’s reality. the numbers report on the prevalence of women who have undergone FGM many years ago. The outcomes of the existing interventions carried on the young population, e.g., education, may show its fruits years later for which is important a closer monitoring to evidence the results of the interventions [28]. Future studies could explore further into every category and country for advancing our knowledge on effective interventions that do not focus on the pre-and post-test designs but on the effectiveness in the reduction in the practice of FGM [4]. Despite these limitations, the study has revealed important insights for FGM and policymaking.

## Conclusion

There is a need to eliminate FGM. Findings indicate that some countries show a declining trend over time while others not. It can be observed that there is heterogeneity and homogeneity in the FGM prevalence within and between countries which may indicate inequality that deserves further investigation. There is considerable economic burden due to delays in the implementation of interventions to reduce or eliminate FGM. These insights can help in the preparation of public health interventions.

### Electronic supplementary material

Below is the link to the electronic supplementary material.

Supplementary Material 1

## Data Availability

All data is available in the public domain and can be downloaded from UNICEF Data and FGM reports. https://data.unicef.org/topic/child-protection/female-genital-mutilation.

## Footnotes

1 The FGM Overview Report, presents a table with single estimates of FGM prevalence for years within the range of 1984 and 1995. For this manuscript, we included this dataset for year 1990.

2 The Current health expenditure per capita, PPP (current international $) is 142.98 dollars per capita per year. This is calculated with an average of the neighbor countries as it does not have its own published. Ethiopia 82.3, Kenya 203.23, Djibouti 105.22, all are year 2020 and obtained in the World Bank statistics database.

## Abbreviations

CAGR: Compound annual growth rate
FGM: Female genital mutilation
SLR: Systematic Literature Review
SDG: Sustainable Development Goals
WHO: World Health Organization
